# A Real-World, Multicenter, Observational Retrospective Study of Durvalumab After Concomitant or Sequential Chemoradiation for Unresectable Stage III Non-Small Cell Lung Cancer

**DOI:** 10.3389/fonc.2021.744956

**Published:** 2021-09-28

**Authors:** Alessio Bruni, Vieri Scotti, Paolo Borghetti, Stefano Vagge, Salvatore Cozzi, Elisa D’Angelo, Niccolò Giaj Levra, Alessandra Fozza, Maria Taraborrelli, Gaia Piperno, Valentina Vanoni, Matteo Sepulcri, Marco Trovò, Valerio Nardone, Elisabetta Lattanzi, Said Bou Selman, Federica Bertolini, Davide Franceschini, Francesco Agustoni, Barbara Alicja Jereczek-Fossa, Stefano Maria Magrini, Lorenzo Livi, Frank Lohr, Andrea Riccardo Filippi

**Affiliations:** ^1^ Radiotherapy Unit, Department of Oncology and Hematology, University Hospital of Modena, Modena, Italy; ^2^ Department of Oncology, Radiation Therapy Unit, Careggi University Hospital, Florence, Italy; ^3^ Radiation Oncology Department, Spedali Civili and University of Brescia, Brescia, Italy; ^4^ Department of Radiation Oncology, Istituto di Ricovero e Cura a Carattere Scientifico (IRCCS) Policlinico San Martino, Genova, Italy; ^5^ Radiation Therapy Department, Arcispedale di Santa Maria Nuova IRCCS (Istituto di Ricovero e Cura a Carattere Scientifico), Reggio Emilia, Italy; ^6^ Advanced Radiation Oncology Department, Istituto di Ricovero e Cura a Carattere Scientifico (IRCCS), Sacro Cuore Don Calabria Hospital, Verona, Italy; ^7^ Radiation Oncology Department, SS. Annunziata Hospital, “G. D’Annunzio” University of Chieti, Chieti, Italy; ^8^ Division of Radiotherapy, European Institute of Oncology (IEO), Istituto di Ricovero e Cura a Carattere Scientifico (IRCCS) European Institute of Oncology, Milan, Italy; ^9^ Radiation Oncology Department, S. Chiara Hospital, Trento, Italy; ^10^ Radiation Oncology Unit, Veneto Institute of Oncology Istituto Oncologico Veneto (IOV), Istituto di Ricovero e Cura a Carattere Scientifico (IRCCS), Padua, Italy; ^11^ Radiation Oncology Department, Azienda Sanitaria Universitaria Integrata, Udine, Italy; ^12^ Radiotherapy Unit, “Ospedale del Mare”, Naples, Italy; ^13^ Radiotherapy Unit, University Hospital of Parma, Parma, Italy; ^14^ Department of Radiotherapy, Bolzano Hospital, Bolzano, Italy; ^15^ Medical Oncology Unit, Department of Oncology and Hematology, University Hospital of Modena, Modena, Italy; ^16^ Department of Radiotherapy and Radiosurgery, Humanitas Research Hospital, Istituto di Ricovero e Cura a Carattere Scientifico (IRCCS)–Humanitas Research Hospital, Milan, Italy; ^17^ Medical Oncology, Fondazione Istituto di Ricovero e Cura a Carattere Scientifico (IRCCS) Policlinico San Matteo, Pavia, Italy; ^18^ Department of Oncology and Hemato-Oncology, University of Milan, Milan, Italy; ^19^ Department of Radiation Oncology, Fondazione Istituto di Ricovero e Cura a Carattere Scientifico (IRCCS) Policlinico San Matteo and University of Pavia, Pavia, Italy

**Keywords:** chemoradiotherapy, immunotherapy, stage III, unresectable, NSCLC

## Abstract

**Introduction:**

For unresectable stage III non-small cell lung cancer (NSCLC), the standard therapy consists of chemoradiotherapy (CRT) followed by durvalumab maintenance for responding patients. The present study reports on the safety and outcome of durvalumab use after CRT in a real-world, multicenter, retrospective cohort.

**Methods:**

Two hundred thirty-eight patients have been included. We collected data on systemic therapy, radiation therapy, the timing between CRT and durvalumab, number of durvalumab cycles, reasons for non-starting or discontinuation, incidence and grade of adverse events (AEs), and progression-free survival (PFS) and overall survival (OS).

**Results:**

One hundred fifty-five patients out of 238 (65.1%) received at least one durvalumab dose: 91 (58.7%) after concomitant CRT (cCRT) and 64 (41.3%) after sequential CRT (sCRT). Programmed-death ligand 1 (PD-L1) status was unknown in 7/155 (4.5%), negative in 14 (9.1%), and positive ≥1% in 134/155 (86.4%). The main reasons for non-starting durvalumab were progression (10.1%), PD-L1 negativity (7.5%), and lung toxicity (4.6%). Median follow-up time was 14 months (range 2–29); 1-year PFS and OS were 65.5% (95%CI: 57.6-74.4) and 87.9% (95%CI: 82.26.6-93.9), respectively. No significant differences in PFS or OS were detected for cCRT vs. sCRT, but the median PFS was 13.5 months for sCRT vs. 23 months for cCRT. Potentially immune-related AEs were recorded in 76/155 patients (49.0%). Pneumonitis was the most frequent, leading to discontinuation in 11/155 patients (7.1%).

**Conclusions:**

Durvalumab maintenenace after concurrent or sequential chemoradiation for unresectable, stage III NSCLC showed very promising short-term survival results in a large, multicenter, restrospective, real-world study. Durvalumab was the first drug obtaining a survival benefit over CRT within the past two decades, and the present study contributes to validating its use in clinical practice.

## Introduction

The randomized phase 3 PACIFIC trial established a new standard for unresectable stage III non-small cell lung cancer (NSCLC), introducing immunotherapy maintenance with the anti-programmed-death ligand 1 (anti-PD-L1) agent durvalumab after chemoradiotherapy (CRT). The use of durvalumab substantially improved both progression-free survival (PFS) and overall survival (OS) in patients responding to CRT (1–3). The subsequent registration and clinical use of durvalumab varied across countries. According to the European Medicines Agency (EMA) recommendations, durvalumab use was approved in Italy in September 2018, restricted to patients with a PD-L1 tumor proportion score (TPS) >1%, following a post-hoc analysis showing that patients with tumors expressing PD-L1 below 1% lacked any survival advantage over control.

As unresectable stage III NSCLC presents heterogeneous clinical features, the therapeutic approach may vary widely across centers. Therefore, in this study, we aimed to describe the use of durvalumab in a real-life context, on a multicenter basis, assessing the adherence to EMA indications and providing information on patients’ demographics, treatment tolerance, and survival.

## Material and Methods

### Study Population and Outcome Assessment

In June 2020, we invited Italian Centers participating in the Association of Radiotherapy and Clinical Oncology (AIRO) thoracic oncology network to include in this observational study all stage III patients referred to radiotherapy, which would have been candidates for CRT and durvalumab after approval in Italy from September 2018 to March 2020. Sixteen centers agreed, for a total of 238 enrolled patients.

Patient demographics, tumor characteristics, and treatment-related information were collected in a centralized digital database. According to standard practice, during durvalumab administration and subsequent follow-up, restaging with total body CT scan was performed every 3 months during the first 2 years and then every 6 months, with variations according to each institution’s preference.

We defined locoregional relapse as either local (primary tumor) or mediastinal failure, while systemic progression as the occurrence of extra-thoracic visceral or nodal metastases. OS probability was calculated from the end of CRT to death for any cause (or last assessment of vital status); PFS was calculated from the end of CRT to any disease progression (local failure and distant progression) or death.

All adverse events (AEs) were categorized using Common Terminology Criteria for Adverse Events (CTCAE) version 4.0.

The Ethical Committee of the Coordinating Center in Modena first reviewed and approved the study (approval number 59/2021/OSS/AOUMO) and then each participating center.

### Statistical Analysis

The univariate Cox proportional-hazards models were performed to screen the effect of the clinical and demographic variables on the PFS and OS. The hazard ratios associated with the PFS and OS were calculated with their 95% confidence interval for each factor from the Cox proportional-hazards model. Those covariates with a p-value <0.05 were then selected for the multivariate analysis, where the PFS and OS were the dependent variable. Multivariate analysis was performed using again the Cox proportional-hazards model.

The likelihood ratio test was used as a test of statistical significance, and the multiple comparisons correction was not performed. Differences, with a p-value less than 0.05, were selected as significant, and data were acquired and analyzed in R v4.0.3 software environment.

## Results

At the end of CRT, 83/238 (34.8%) patients did not start durvalumab. The main reasons were the absence of PD-L1 expression (n = 18; 7.5%), persistent CRT-related toxicities (n = 14; 5.9%), disease progression (n = 24; 10%), denial of consent (n = 2; 0.8%), death due to other causes (n = 2; 0.8%), viral infections (n = 5; 2.2%, including SARS-CoV-2), and acute renal injury (n = 1; 0.4%). The remaining 17 patients (7.2%) did not start durvalumab due to unknown causes.

One hundred fifty-five out of 238 patients (65.2%) received at least one durvalumab dose after CRT. Main patients and tumors characteristics are reported in **Table 1**; 150/155 (95.5%) underwent fluorine-18-deoxyglucose positron emission tomography (^18^FDG-PET) for staging and 141 (90.9%) brain CT scan or MRI. One hundred twelve patients (77.5%) received 60 Gray (Gy) in 30 fractions, 10 (6.5%) received 66 Gy in 33 fractions, and 10 (6.5%) received 44–54 Gy in 22–27 fractions; 15 patients (9.5%) received 51–55 Gy in 17–20 fractions. All patients received platinum-based chemotherapy, mostly weekly carboplatin/taxanes (33.5%), and platinum/etoposide every 3 weeks (20.6%).

**Table 1:** Patients’ characteristics.

Category	Variables	Percentage (%)
Age (mean)	66 (40–82)	
Gender	Male	109 (70.3%)
	Female	46 (29.7%)
Smoking habit	Active smokers	11 (7.1%)
	Former smokers	88 (56.8%)
	Never smokers	56 (36.1%)
Performance status (ECOG)	0	93 (60.0%)
	1	57 (36.8%)
	2	5 (3.2%)
Cardiac comorbidities	Yes	50 (32.2%)
	No	105 (67.8%)
Hypertension	Yes	75 (48.4%)
	No	80 (51.6%)
Histology	Adenocarcinoma	92 (59.3%)
	SCC	49 (31.6%)
	Other	14 (9.1%)
PD-L1 expression	<1%	14 (9.1%)
	1–50%	71 (45.8%)
	>50%	63 (40.6%)
	Unknown	7 (4.5%)
T stage	T1	21 (13.5%)
	T2	46 (29.7%)
	T3	44 (28.4%)
	T4	44 (28.4%)
N stage	N0	4 (2.5%)
	N1	13 (8.4%)
	N2	94 (60.7%)
	N3	44 (28.4%)
TNM staging (9th edition)	IIIA	51 (32.9%)
	IIIB	85 (54.9%)
	IIIC	19 (12.2%)
Chemoradiotherapy	Concomitant	91 (58.8%)
	Sequential	64 (41.2%)

TNM, tumor node metastasis; ECOG, Eastern Cooperative Oncology Group; PD-L1, programmed-death ligand 1.

Concomitant CRT (cCRT) has been administered in 91 patients (58.8%) and sequential CRT (sCRT) in 64 (41.2%). **Table 2** describes patients’ and tumor characteristics of these two subgroups. As expected, those who received sCRT were older, with larger tumors, and more likely to receive hypofractionated RT.

**Table 2:** Patients’ characteristics for concomitant vs. sequential chemoradiotherapy.

Category		Concomitant CRT	Sequential CRT
Patients	Total number	91	64
Age	Mean (range)	64 (40–80)	69 (43–82)
	Median (range)	66 (40–80)	72 (43–82)
Gender	Male (%)	61 (67.1%)	48 (75%)
	Female (%)	30 (32.9%)	16 (25%)
Smoke habit	Active smokers	37 (40.7%)	19 (29.7%)
	Former smokers	48 (52.8%)	40 (62.5%)
	Never smokers	6 (6.5%)	5 (7.8%)
Cardiac comorbidities	No (%)	68 (74.7%)	37 (57.8%)
	Yes (%)	23 (25.3%)	27 (42.2%)
Histology	Adenocarcinoma	51 (56.1%)	41 (64.1%)
	SCC	31 (34.1%)	18 (28.1%)
	Other	9 (9.8%)	5 (7.8%)
PD-L1 expression	<1%	8 (8.7%)	6 (9.3%)
	1%–25%	36 (39.6%)	22 (34.4%)
	25%–50%	3 (3.3%)	10 (15.6%)
	>50%	38 (41.8%)	25 (39.1%)
	Unknown	7 (7.6%)	1 (1.6%)
T stage	T1	16 (17.6%)	5 (7.8%)
	T2	31 (34.1%)	15 (23.4%)
	T3	26 (28.6%)	18 (25.0%)
	T4	18 (19.7%)	26 (40.8%)
N Stage	N0	2 (2.2%)	2 (3.2%)
	N1	8 (8.8%)	5 (7.8%)
	N2	59 (64.8%)	35 (54.7%)
	N3	22 (24.2%)	22 (34.3%)
^18^FDG-PET	Yes	89 (97.8%)	61 (95.3%)
	No	2 (2.2%)	3 (4.7%)
Clinical stage	IIIA	37 (40.6%)	14 (21.9%)
	IIIB	44 (48.3%)	41 (64.1%)
	IIIC	10 (11.1%)	9 (14.0%%)
Chemotherapy cycles	Median (range)	4 (1–9)	4 (3–8)
	Two to three	28 (30.7%)	26 (40.6%)
	Four	22 (24.2%)	29 (45.3%)
	More than four	36 (39.6%)	9 (13.6%)
RT schedule	Conventional	81 (89.1%)	49 (76.5%)
	Hypofractionation	10 (9.9%)	15 (23.5%)
RT total dose	>66 Gy	10 (10.9%)	2 (3.2%)
	60–66 Gy	76 (83.6%)	51 (79.7%)
	<60 Gy	5 (5.5%)	11 (17.1%)
Clinical response	Complete response	4 (4.4%)	2 (3.2%)
	Partial response	71 (78.1%)	45 (70.3%)
	Stable disease	14 (15.3%)	16 (25.0%)
	Unknown	2 (2.2%)	1 (1.5%)
Time interval CRT—durvalumab first dose	Days (range)	56 (10–245)	51 (9–153)

Twenty-two percent of patients started durvalumab <42 days from CRT and 78% after 42 days. The median time from CRT to first durvalumab dose was 52 days (range 9–245).

At the time of writing, 54 of 155 (35.4%) patients were still on treatment. The main reasons for durvalumab discontinuation are reported in **Table 3**. The mean and median numbers of durvalumab cycles were 14 and 13 (range 1–34), respectively.

**Table 3:** Reasons for treatment discontinuation.

Reasons for durvalumab discontinuation	n. of patients (%)
Achieving planned total dose as per PACIFIC study	36 (23.4%)
Disease progression	35 (22.6%)
Pneumonitis	11 (7.1%)
Diarrhea	4 (2.6%)
Thyroiditis	3 (1.9%)
Cardiovascular disease	3 (1.9%)
Liver dysfunction	2 (1.3%)
Neutropenia	2 (1.3%)
Skin reactions	1 (0.6%)
Pancreatic failure	1 (0.6%)
COVID-19	1 (0.6%)
Other	2 (1.3%)
Total	101/155 (65.2%)

Covid19, Coronavirus Disease 2019.

### Survival

**Figure 1:**
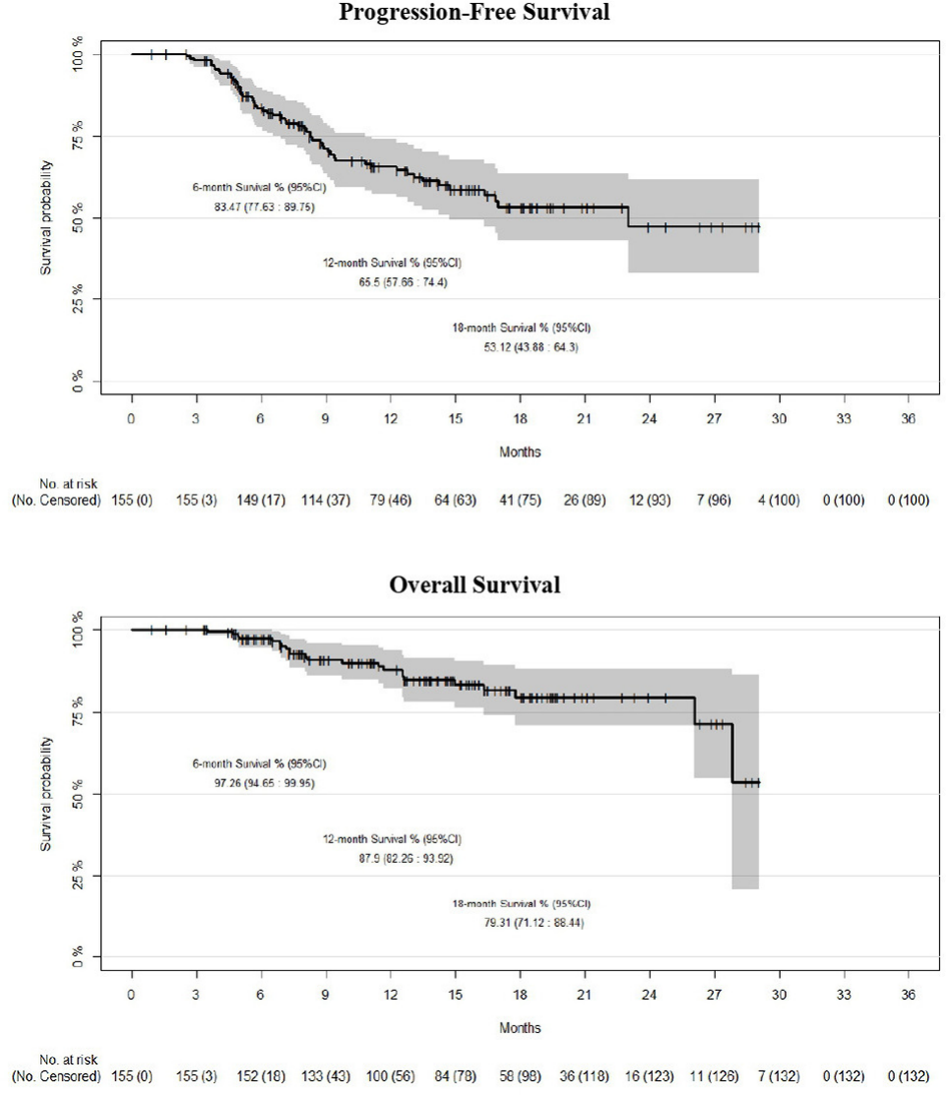
Kaplan-Meier survival estimates (progression-free and overall survival) for the whole cohort (n = 155).

PFS at 6, 12, and 18 months was 83.5% (95%CI: 77.6–89.7), 65.5% (95%CI: 57.6–74.4), and 53.1% (95%CI: 43.8–64.3), respectively (**Figure 1**). OS at 6, 12, and 18 months was 97.2% (95%CI: 94.6– 99.9), 87.9% (95%CI: 82.26–93.9), and 79.3% (95%CI: 71.1–88.4), respectively (**Figure 1**). Median PFS was 23 months, and median OS was not reached.

**Figure 2:**
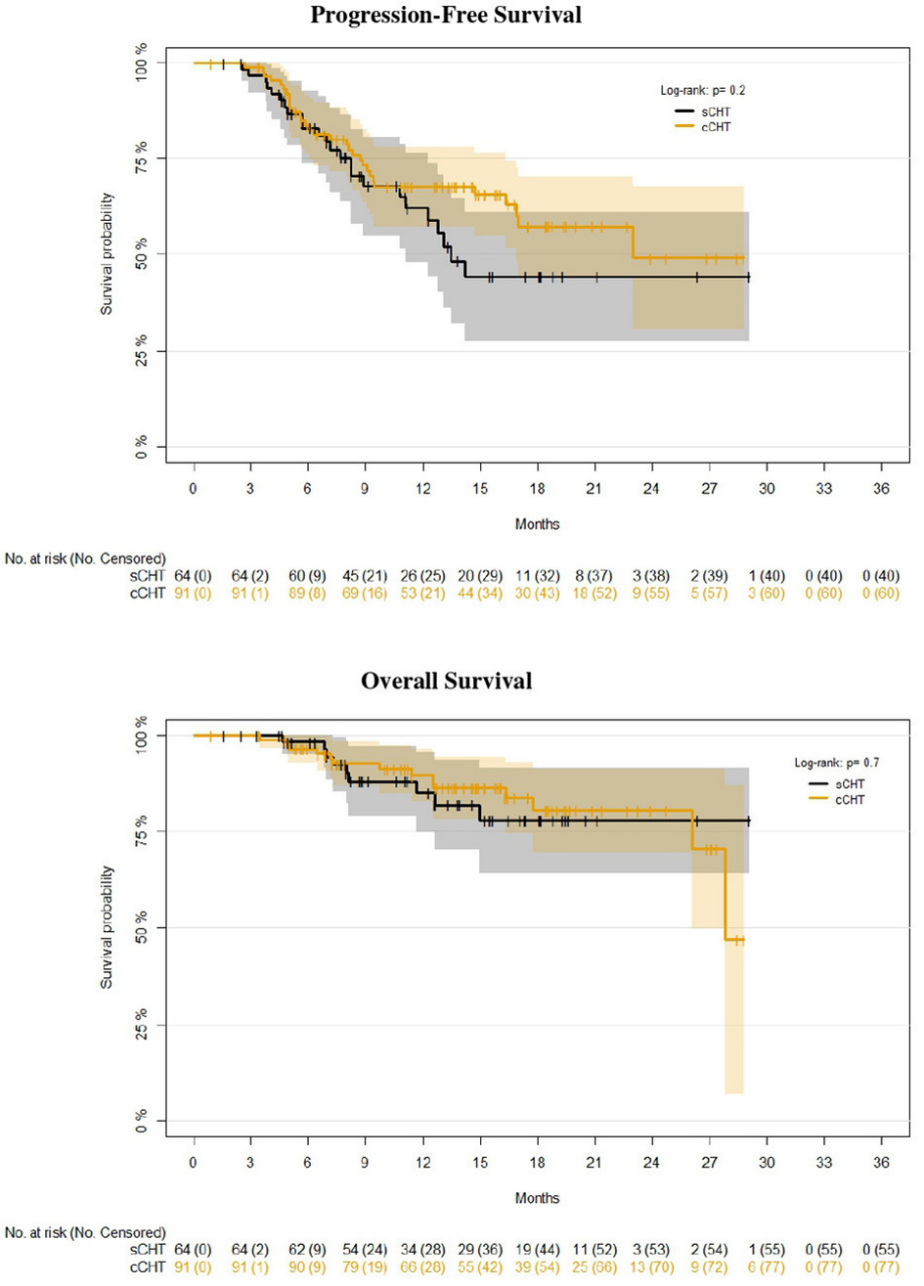
Kaplan–Meier survival estimates (progression-free and overall survival) of concurrent (n = 91) vs. sequential (n = 64) chemoradiotherapy (cCRT vs. sCRT).

We did not detect any significant difference in PFS (log-rank p = 0.2) or OS (log-rank p = 0.7) between concurrent and sCRT plus durvalumab (**Figure 2**). However, the median PFS was 13.5 and 23.0 months for sCRT and cCRT, respectively.

The univariate analysis demonstrated a significant association among TNM staging, histology, and PFS (p-value ≤ 0.05). The multivariate analysis confirmed a statistically significant effect of staging and histology on PFS (p-values: 0.022 and 0.016, respectively). In particular, the risk of progression was about 2.5 times more likely in patients with stage IIIC vs. IIIA, keeping constant histology (HR = 2.53). In addition, the risk of progression was about 1.9 times more likely in patients with squamous cell carcinoma (SCC) vs. non-squamous histology, maintaining constant TNM staging (HR = 1.92).

A significant association between histology and OS was also observed (p-values = 0.039). In particular, the risk of death was about 2.4 times more likely in patients with SCC vs. non-squamous histology (HR = 2.39).

### Pattern of Relapse

At the time of writing, 55 patients (35.5%) relapsed locally or systemically; 32 (20.6%) had locoregional progression [as the only site of disease progression in nine (5.8%)], and 46 (29.7%) developed systemic metastases; 23 patients (14.8%) had both local and systemic relapse, and 23 (14.8%) had systemic relapses alone. Primary metastatic sites were the brain, lung (ipsilateral and contralateral), liver, and bone. More than one metastatic site was found in 17 patients. Nine patients had less than five metastatic sites (5/46, 10.8%), while 37/46 (89.2%) experienced poly-metastatic spread.

At progression, 30/55 patients (54.5%) received systemic treatment (26 with chemotherapy and four with pembrolizumab), and nine (16.3%) received metastasis-directed stereotactic RT (exclusive salvage in five patients at the time of analysis). The remaining 16 patients (29%) were referred to palliative care.

### Toxicity

We defined AEs occurring before the first durvalumab dose as CRT-related, while all other AEs were defined as potentially immune-related. We report in details AEs recorded in patients receiving at least one durvalumab dose (n = 155). One hundred five patients experienced at least one AE related to CRT administration. Grade 1–2 esophagitis was the most common (80/155, 50.3%), followed by grade 1–2 lung toxicity recorded in 36/155 patients (23.2%). In comparison, only three patients experienced grade 3 pneumonitis (1.9%). Hematological toxicity was recorded in 13 patients (four patients with grade 2, two with grade 3, and two with grade 4).

Potentially immune-related AEs (defined as a “side effect occurred after at least one cycle of durvalumab and not previously reported”) were recorded in 76/155 patients (49.0%). The most frequent were pneumonitis (27/155, 17.4%; 85.2% of whom were G1-2, 11.1% G3, and 3.7% G4), causing definitive discontinuation of durvalumab in 11 patients (7.1%) and then myalgia/asthenia (27/155, 17.4%; all were G1–G2) and thyroiditis (11/155, 7.1%; of whom 91.9% were G1–G2 and 9.1% were G3). We report all reasons for durvalumab permanent discontinuation in **Table 2**.

## Discussion

We report the findings of a multicenter, observational, retrospective study in patients with unresectable stage III NSCLC candidates to CRT and durvalumab outside clinical trials or expanded access programs (EAPs).

A consistent proportion of the whole cohort (n = 83; 34.8%) did not receive durvalumab after CRT, primarily for PD-L1 negativity or CRT-related toxicity. For those receiving at least one durvalumab dose (n = 155), demographic characteristics were quite similar to the PACIFIC trial, with a median age of 66 but a prevalence of PD-L1-positive patients (86.4%) and a higher rate of stage IIIB or IIIC (67.1%, **Table 1**). Considering the limited follow-up, PFS and OS are slightly higher than in PACIFIC trial^1,2,3^, and in line with the very positive findings of previously published observational series (4–6) including comparisons with historical cohorts treated in the pre-immunotherapy era. In the most extensive retrospective series reported so far, including 147 patients treated with durvalumab after concurrent CRT, from Canadian and Japanese Centers (6), 12 months’ OS was above 90%, reaching 100% for patients affected with tumors expressing PD-L1 >50%. The median time to durvalumab first dose was 33 days, relatively short for a real-life study; no impact on survival was detected for patients initiating durvalumab >42 days after CRT.

In our study, 12-month PFS was 65.5%, and OS 87.9%, and the crude rate of local failure was 21%. In assessing these values, we should take into account the methodological limitations of any direct comparison between different study designs and patient populations and, in particular, the uncertainties in PFS assessment (influenced by the absence of a clear follow-up protocol and RECIST use in our study), which might lead to PFS overestimation.

A detailed analysis of the pattern of relapse suggested that most of the patients relapsed outside the thorax, many with poly-metastatic disease (60.9%). This latter finding is partially in contrast with the PACIFIC trial, in which intrathoracic progression was the most common compared with metastatic progression (80.6% vs. 15.3%, respectively, in the durvalumab arm, 74.5% vs. 20.3%, respectively, in the placebo arm) (7). At the time of progression, 16.3% of patients did receive local therapy alone and 54.5% chemotherapy. These preliminary data, which need to be confirmed by larger observational series, are relevant for the design of future clinical trials dedicated to progressors after CRT plus durvalumab.

Of particular interest with this series is the inclusion of patients treated with sCRT, who were eligible for durvalumab maintenance according to EMA indications. They represent a meaningful proportion of our cohort (41.2%), reflecting national preferences. We found no significant differences in PFS or OS for these patients when compared with patients receiving concurrent CRT; however, median PFS was remarkably higher for patients receiving concurrent CRT (23 vs. 13.5 months), and this result is mainly due to progressions after the first 12 months from RT (taking into account the low number of patients at risk and events, with related statistical uncertainties). These are probably the most interesting and novel findings of this study, suggesting that the two approaches (cCRT or sCRT) might achieve similar survival rates (especially OS), despite some differences in patients and tumor characteristics (**Table 2**), but with different median PFS. Notably, PFS and OS were calculated from the end of RT, with the aim of comparing these data with the PACIFIC survival data, which have been calculated from the date of randomization post-CRT.

The prospective ongoing PACIFIC-6 trial (NCT03693300) will better clarify the safety (first objective) and efficacy (secondary objective) of sCRT plus durvalumab, and additional information is expected from the publication of the survival data of the PACIFIC-R real-world study (NCT03798535). However, in the PACIFIC-R study, only 14% of patients received sCRT (based on EAP data). Notably, the median time interval between RT and durvalumab first dose was 52 days, as reported for our cohort (8).

At multivariate analysis, we found that patients affected with SCC are more likely to progress and die, confirming what was already shown by other studies (6).

The safety profile was in general analogous to other series, with the most common cause for durvalumab discontinuation being pneumonitis (7.1%) (1–6). Many research strategies are currently being investigated to further improve the outcomes of CRT and immunotherapy combinations (9); the present study results, together with similar findings, support the feasibility and the translation to practice of phase 3 trials’ results in this particular setting.

The strength points of the study are as follows: a) a very homogeneous cohort of patients affected by stage III PD-L1 >1% NSCLC; b) to our knowledge, the first study reporting the main reasons why durvalumab was not started after CRT; and c) the results of sCRT showed from a real world series. On the other hand, the main limitations of our study are represented by its retrospective nature and, secondly, by the relatively short follow-up time, which may influence survival projections in the mid-term to long term.

In conclusion, maintenance therapy with durvalumab, for stage III unresectable NSCLC, PD-L1 >1%, responding to cCRT or sCRT, was associated with very promising short-term survival rates in a large multicenter, retrospective, real-world series. Durvalumab was confirmed to be the first drug obtaining a survival benefit over CRT within the past two decades, and the present study contributes to validating its use in clinical practice.

## Data Availability Statement

The original contributions presented in the study are included in the article/supplementary material. Further inquiries can be directed to the corresponding author.

## Ethics Statement

The studies involving human participants were reviewed and approved by the Ethical Committee of “Area Vasta Emilia Nord”—University Hospital of Modena. The patients/participants provided their written informed consent to participate in this study.

## Author Contributions

AB, VS, PB, SV, and ARF: conceptualization. AB, SC, ED’A, NG, AF, MTa, GP, VV, MS, MTr, VN, EL, SS, FB, DF, and FA: data curation. AB and ARF: formal analysis. AB, ARF, and FL: original draft writing. AB, ARF, BJ-F, SM, LL, and FL: review and editing. All authors contributed to the article and approved the submitted version.

## Conflict of Interest

AB: speakers’ bureau from Astra Zeneca, MSD, Tecnologie Avanzate; and advisory role for Astra Zeneca. VS: speakers’ bureau for Astra Zeneca, Roche, Accuray Int.; and advisory role for Astra Zeneca. PB: speakers’ bureau for Astra Zeneca. SV: speakers’ bureau for Astra Zeneca, Roche, Accuray Int. ED’A: speaker’s bureau from Nestlè, MSD, Astra Zeneca; travel expenses: IPSEN; and expert testimony: Nestlè. NG: speakers’ bureau for Astra Zeneca. DF: speakers’ bureau for Astra Zeneca; and advisory role for AstraZeneca. FA: speakers’ bureau for Astra Zeneca, Roche, BMS, Boehringer Ingelheim, MSD; and advisory role for Boehringer Ingelheim, MSD. BJ-F: personal fees from Janssen, Roche, Astra Zeneca, and Accuray, and institutional grants from AIRC, FIEO-CCM and Accuray Int., all outside of the submitted work. ARF: speakers’ bureau for Astra Zeneca, MSD, Roche, Ipsen; advisory role for Astra Zeneca, Roche; institutional research funding: Astra Zeneca; and honoraria for study conduction (not related to the present study): Astra Zeneca.

The remaining author declares that the research was conducted in the absence of any commercial or financial relationships that could be construed as a potential conflict of interest.

## Publisher’s Note

All claims expressed in this article are solely those of the authors and do not necessarily represent those of their affiliated organizations, or those of the publisher, the editors and the reviewers. Any product that may be evaluated in this article, or claim that may be made by its manufacturer, is not guaranteed or endorsed by the publisher.
