# Corrigendum: A Real-World, Multicenter, Observational Retrospective Study of Durvalumab After Concomitant or Sequential Chemoradiation for Unresectable Stage III Non-Small Cell Lung Cancer

**DOI:** 10.3389/fonc.2021.802949

**Published:** 2021-11-16

**Authors:** Alessio Bruni, Vieri Scotti, Paolo Borghetti, Stefano Vagge, Salvatore Cozzi, Elisa D’Angelo, Niccolò Giaj Levra, Alessandra Fozza, Maria Taraborrelli, Gaia Piperno, Valentina Vanoni, Matteo Sepulcri, Marco Trovò, Valerio Nardone, Elisabetta Lattanzi, Said Bou Selman, Federica Bertolini, Davide Franceschini, Francesco Agustoni, Barbara Alicja Jereczek-Fossa, Stefano Maria Magrini, Lorenzo Livi, Frank Lohr, Andrea Riccardo Filippi

**Affiliations:** ^1^ Radiotherapy Unit, Department of Oncology and Hematology, University Hospital of Modena, Modena, Italy; ^2^ Department of Oncology, Radiation Therapy Unit, Careggi University Hospital, Florence, Italy; ^3^ Radiation Oncology Department, Spedali Civili and University of Brescia, Brescia, Italy; ^4^ Department of Radiation Oncology, Istituto di Ricovero e Cura a Carattere Scientifico (IRCCS) Policlinico San Martino, Genova, Italy; ^5^ Radiation Therapy Department, Arcispedale di Santa Maria Nuova IRCCS (Istituto di Ricovero e Cura a Carattere Scientifico), Reggio Emilia, Italy; ^6^ Advanced Radiation Oncology Department, Istituto di Ricovero e Cura a Carattere Scientifico (IRCCS), Sacro Cuore Don Calabria Hospital, Verona, Italy; ^7^ Radiation Oncology Department, SS. Annunziata Hospital, “G. D’Annunzio” University of Chieti, Chieti, Italy; ^8^ Division of Radiotherapy, European Institute of Oncology (IEO), Istituto di Ricovero e Cura a Carattere Scientifico (IRCCS) European Institute of Oncology, Milan, Italy; ^9^ Radiation Oncology Department, S. Chiara Hospital, Trento, Italy; ^10^ Radiation Oncology Unit, Veneto Institute of Oncology Istituto Oncologico Veneto (IOV), Istituto di Ricovero e Cura a Carattere Scientifico (IRCCS), Padua, Italy; ^11^ Radiation Oncology Department, Azienda Sanitaria Universitaria Integrata, Udine, Italy; ^12^ Radiotherapy Unit, “Ospedale del Mare”, Naples, Italy; ^13^ Radiotherapy Unit, University Hospital of Parma, Parma, Italy; ^14^ Department of Radiotherapy, Bolzano Hospital, Bolzano, Italy; ^15^ Medical Oncology Unit, Department of Oncology and Hematology, University Hospital of Modena, Modena, Italy; ^16^ Department of Radiotherapy and Radiosurgery, Humanitas Research Hospital, Istituto di Ricovero e Cura a Carattere Scientifico (IRCCS)–Humanitas Research Hospital, Milan, Italy; ^17^ Medical Oncology, Fondazione Istituto di Ricovero e Cura a Carattere Scientifico (IRCCS) Policlinico San Matteo, Pavia, Italy; ^18^ Department of Oncology and Hemato-Oncology, University of Milan, Milan, Italy; ^19^ Department of Radiation Oncology, Fondazione Istituto di Ricovero e Cura a Carattere Scientifico (IRCCS) Policlinico San Matteo and University of Pavia, Pavia, Italy

**Keywords:** chemoradiotherapy, immunotherapy, stage III, unresectable, NSCLC

In the original article there was an error. **The survival numbers were incorrect.**

A correction has been made to ***Abstract:***

**“1-year PFS and OS were 83.5% (95%CI: 77.6-89.7) and 97.2% (95%CI: 94.6-99.9), respectively**.”

**“1-year PFS and OS were 65.5% (95%CI: 57.6-74.4) and 87.9% (95%CI: 82.26.6-93.9), respectively”**

In the original article, there was an error. **The survival numbers were incorrect.**

A correction has been made to ***Results, Survival:***

**“PFS at 12, 18, and 24 months was 83.5% (95%CI: 77.6–89.7), 65.5 (95%CI: 57.6–74.4), and 53.1% (95%CI: 43.8–64.3), respectively. (Figure 1). OS at 12, 18, and 24 months was 97.2% (95%CI: 94.6– 99.9), 87.9% (95%CI: 82.26–93.9), and 79.3% (95%CI: 71.1–88.4), respectively (Figure 1)**.”

**“PFS at 6, 12, and 18 months was 83.5% (95%CI: 77.6–89.7), 65.5% (95%CI: 57.6–74.4), and 53.1% (95%CI: 43.8–64.3), respectively. (Figure 1). OS at 6, 12, and 18 months was 97.2% (95%CI: 94.6– 99.9), 87.9% (95%CI: 82.26–93.9), and 79.3% (95%CI: 71.1–88.4), respectively (Figure 1)”**

In the original article, there was an error. **The survival numbers were incorrect.**

A correction has been made to ***Discussion:***

***“12-month PFS was 83.5%, and OS 97.2%”***

***“12-month PFS was 65.5%, and OS 87.9%”***

The authors apologize for these errors and state that this does not change the scientific conclusions of the article in any way. The original article has been updated.

## Publisher’s Note

All claims expressed in this article are solely those of the authors and do not necessarily represent those of their affiliated organizations, or those of the publisher, the editors and the reviewers. Any product that may be evaluated in this article, or claim that may be made by its manufacturer, is not guaranteed or endorsed by the publisher.

